# The Travels of Signet-Ring Cell Carcinoma: From Colon to Stomach and Duodenum

**DOI:** 10.14309/crj.0000000000001239

**Published:** 2023-12-21

**Authors:** Chloe K. Tom, Nicholas Placone, Evan Yung, Anisa Shaker

**Affiliations:** 1 Division of Gastrointestinal and Liver Diseases, University of Southern California, Los Angeles, CA; 2 Department of Pathology, University of Southern California, Los Angeles, CA; 3 Division of Gastrointestinal and Liver Diseases and Swallowing and Esophageal Disorders Center, University of Southern California, Los Angeles, CA

**Keywords:** colorectal cancer, signet-ring cell carcinoma, metastatic cancer, esophagogastroduodenoscopy

## Abstract

Colorectal cancer (CRC) metastasizing to the stomach and duodenum is rare. Even rarer is when the CRC subtype is signet-ring cell carcinoma (SRCC). Endoscopic findings of CRC metastasis to the stomach have been described as solitary and submucosal while duodenal metastasis has been observed to be exophytic. In this report, we describe a case of a middle-aged man with colon SRCC presenting with oral intolerance. He was found to have concurrent metastases to the stomach and duodenum and died 8 months after his SRCC diagnosis.

## INTRODUCTION

Colorectal cancer (CRC) is the fourth most common type of cancer and the second highest cause of cancer deaths in the United States, with over 150,000 expected new CRC diagnoses in 2023.^1,2^ The mortality from CRC remains high, as over one-third of patients die of the disease, with metastatic disease being the leading cause of CRC-related mortality.^2,3^ CRC metastasis to the stomach is rare, with an incidence of around 4.7% of cases.^4^ Even rarer is CRC spreading to the duodenum with only a handful of published case reports.^5^ Different histologic subtypes of CRC may have different metastatic patterns.^6^ Whereas adenocarcinoma typically spreads to the liver or lung, the less common mucinous adenocarcinoma and signet-ring cell carcinoma (SRCC) have more atypical metastatic patterns, frequently affecting the peritoneum and distant lymph nodes.^7^ SRCC is particularly rare, representing approximately 1% of CRC subtypes, and is associated with poor overall survival.^8^ We present the case of a patient with a SRCC type of CRC histology and atypical gastric and duodenal lesions, which were confirmed to be metastatic SRCC on biopsy.

## CASE REPORT

A 55-year-old man with ulcerative colitis, known T1N0M0 colon SRCC of the sigmoid colon with liver metastases, who was recently treated with partial colectomy and palliative chemotherapy presented to the emergency department with fatigue and weight loss because of early satiety, nausea, and vomiting.

Initial blood pressure was 83/51 mm Hg, and heart rate was 111 beats per minute, which improved with fluid resuscitation. On physical examination, he was frail and tachypneic, and his abdomen was distended but nontender. Laboratory tests on admission were remarkable for hemoglobin 7.2 g/dL and acute kidney injury with creatinine 3.3 mg/dL. An abdominal-pelvic computed tomography scan showed diffuse colonic wall thickening, moderate ascites, numerous hepatic lesions consistent with his known metastatic disease, and a fluid and gas-filled stomach. Over 500cc of gastric secretions were removed with a nasogastric tube, and paracentesis was performed with cytology showing adenocarcinoma with signet-ring cell morphology of gastrointestinal origin.

**Figure 1.**
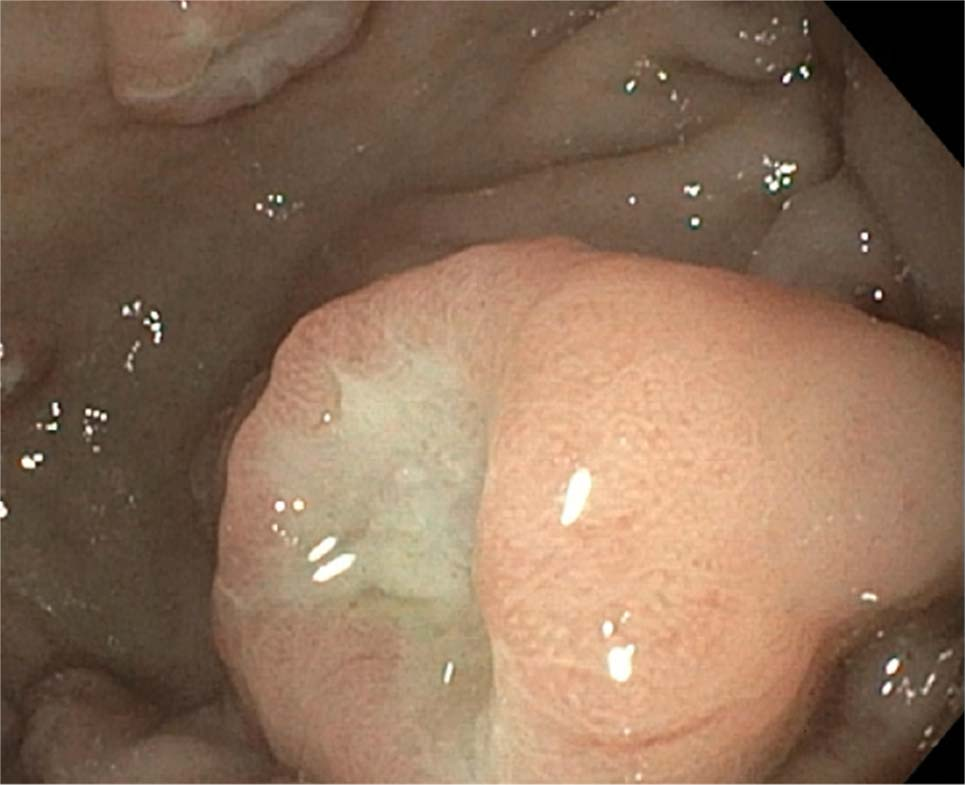
Esophagogastroduodenoscopy reveals polypoid lesion with central ulceration in the antrum and anterior and posterior walls of the gastric body.

**Figure 2.**
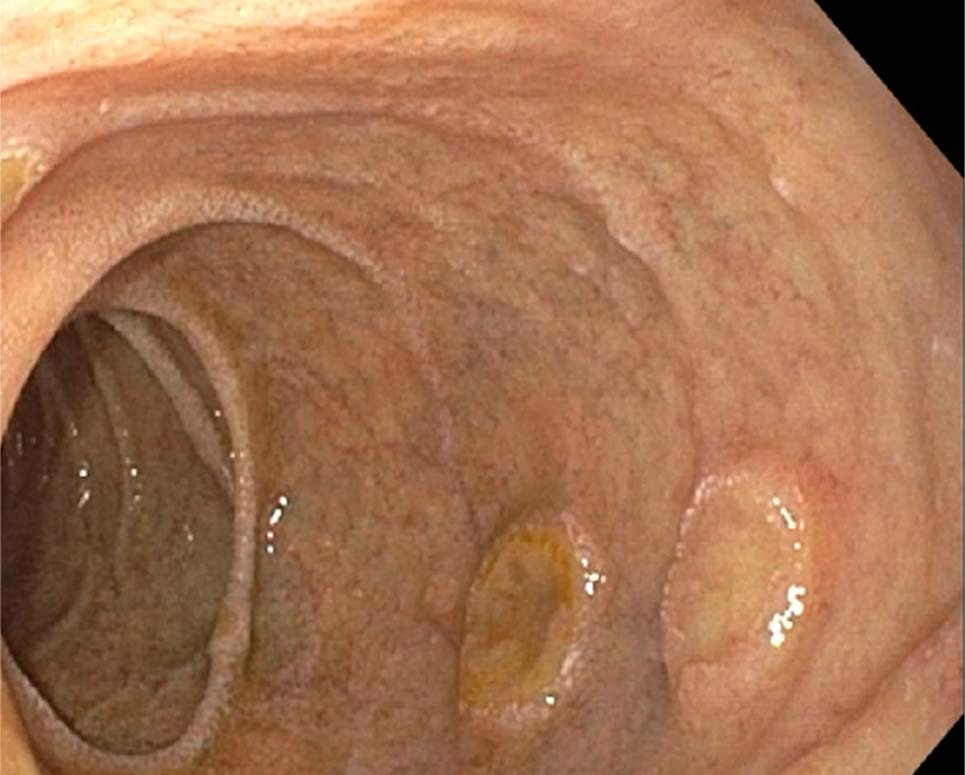
Esophagogastroduodenoscopy reveals multiple raised coin-shaped lesions with central ulceration in the duodenal bulb and second portion of the duodenum.

**Figure 3.**
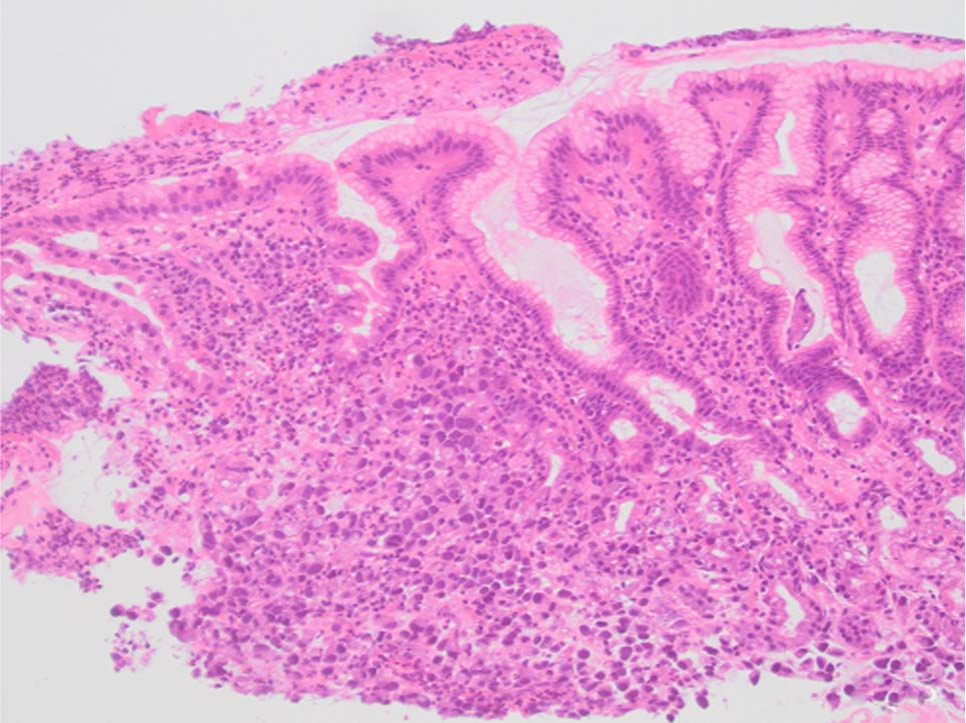
Gastric biopsy showing effacement of gastric architecture by malignancy (H&E, 100×).

**Figure 4.**
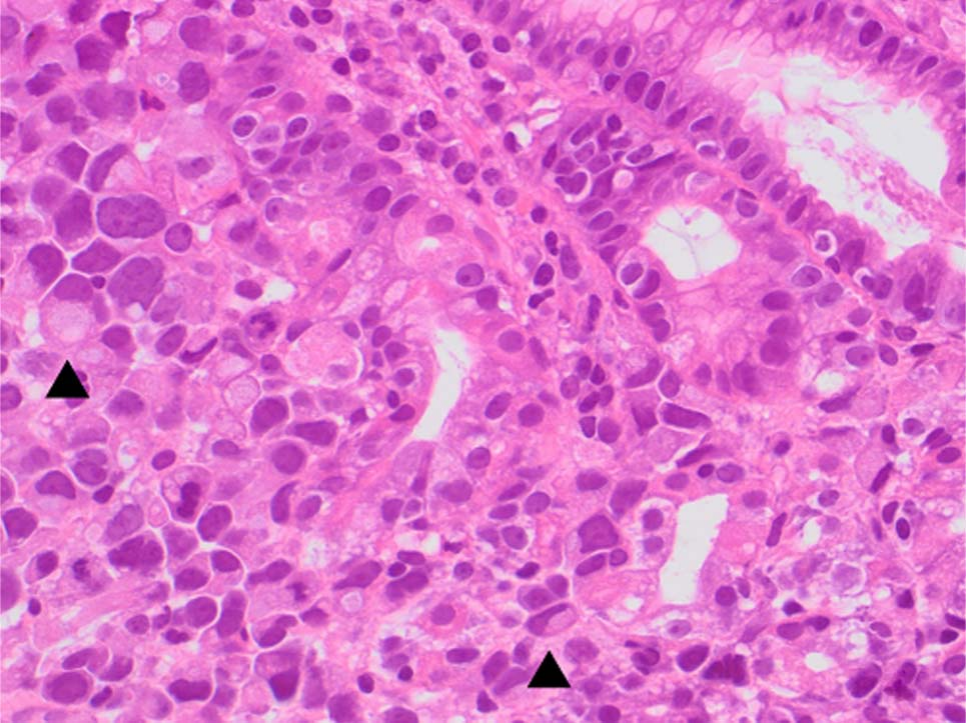
Higher magnification of the previous figure, showing an oxyntic-type gland (center) surrounded and infiltrated by tumor cells. Many cells contain a large mucin vacuole, imparting a signet-ring morphology (arrowhead) (H&E, 400×).

**Figure 5.**
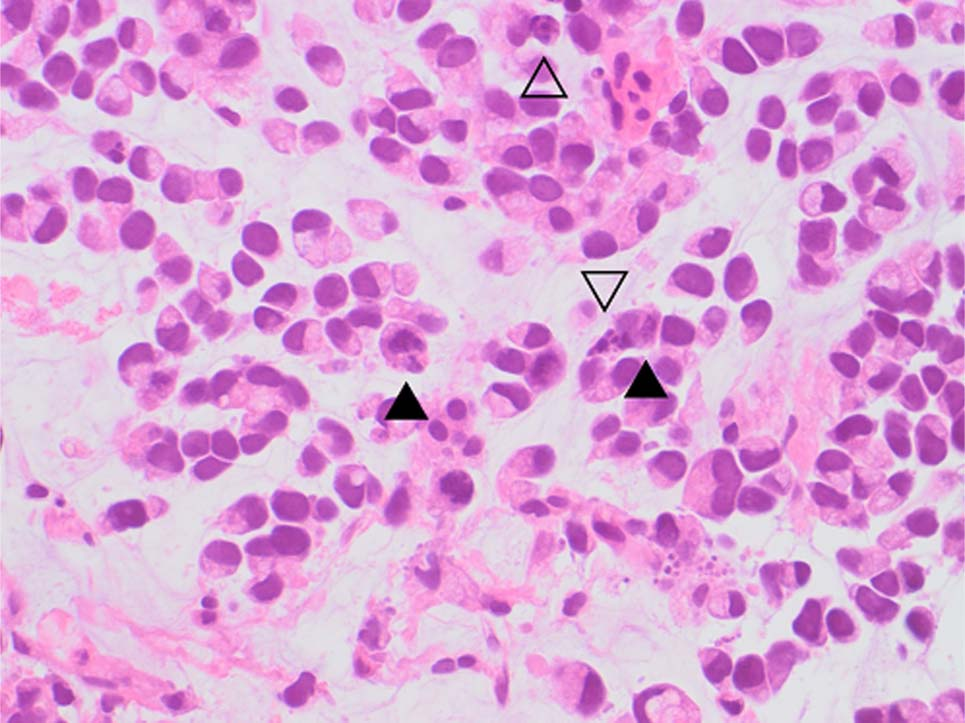
High magnification of the duodenal biopsy. Tumor cells are histologically like those seen in gastric biopsy. Mitotic figures (solid arrowhead) and apoptotic bodies (hollow arrowhead) are readily identified (H&E, 400×).

He underwent esophagogastroduodenoscopy, which revealed an edematous pylorus in addition to numerous raised coin-shaped lesions with central ulceration in the gastric antrum, along the anterior and posterior walls of the gastric body (Figure 1), and the first and second portions of the duodenum (Figure 2). The pathology of the gastric (Figures 3 and 4) and duodenal (Figure 5) lesions showed poorly differentiated adenocarcinoma with signet-ring cell features suggesting colon cancer metastasis to the duodenum and stomach.

The patient opted for hospice care because of widespread metastatic disease and multiple failed chemotherapy regimens. A palliative pyloric stent was placed, and he died a few weeks after discharge.

## DISCUSSION

Although gastric metastasis from primary CRC is uncommon, based on a series of case reports, endoscopic appearance is typically a solitary lesion resembling a submucosal tumor, fungating lesion, or polypoid masses with erosion, or ulceration.^9–12^ There is minimal literature describing CRC spreading to the duodenum, although 1 case described an exophytic mass covering 75% of the second portion of the duodenum circumference without obstruction.^5^

While gastric metastasis of CRC has been described, we identified only 1 case report that described the SRCC subtype extending from the colon to the stomach.^13^ In this instance, esophagogastroduodenoscopy revealed a 2 cm submucosal lesion in the gastric body and lesser curvature with a smooth surface, central depression, and erosion. We also found 1 report where SRCC of the duodenum spread to the ovaries, peritoneum, and colon, and on endoscopy, there was an exophytic obstructing duodenal bulb mass.^14^

SRCC is a unique histologic subtype of CRC and is defined as a poorly cohesive carcinoma^15^ where greater than 50% of tumor cells show abundant cytoplasm mucin and eccentric crescent-shaped nuclei.^16^ SRCC in the distal gastrointestinal tract comprises 1% of all cases of colon cancer and 15.3% of all SRCC cases.^15^ It is hypothesized that the loss of cell-cell adhesion and mucin accumulation contribute to its aggressive carcinogenesis and poor prognosis.^17^

One large epidemiological study found that SRCC was most found in White patients (75% of cases), without gender predilection, and that incidence has declined since 2000 with improvements in CRC screening.^18^ The most common initial stage of diagnosis was stage IV, usually in the proximal colon and often with multiple metastatic sites.^6^ The metastatic pattern of SRCC of the colon is unique in its propensity to spread to the peritoneum as opposed to the liver, as is most common in colonic adenocarcinoma.^6^ In contrast to other colon cancer histologic types, patients younger than 35 years seem to have worse outcomes when diagnosed with SRCC of the colon compared with patients older than 35 years.^19^ Owing to therapeutic advancements in the treatment of SRCC, including surgical resection, adjuvant chemotherapy, and radiotherapy over the past decade, overall survival for SRCC has slightly improved, but still has an unfavorable prognosis.^18^

Fortunately, research groups are analyzing the molecular characteristics of SRCC to develop tailored therapy. Using next-generation sequencing, a unique profile of genes and biomarkers, including Kristen Rat Sarcoma Viral oncogene homolog wild-type (wt), PIK3CA wt, TP53, AT-rich interaction domain 1A, and CDH1, have been identified in SRCC compared with those of adenocarcinoma.^8,20^ Investigations of potential therapeutics include the role of mucin proteins to selectively target SRCC^21^ and clinical trials targeting a promising biomarker, Claudin18.2 (CLDN18.2).^22^

Our case is the first to report colon SRCC metastasizing to the stomach and duodenum with raised coin-shaped lesions with central depression and erosion. The aggressive SRCC histopathology and multiple-site metastatic spread portend a poor prognosis. Our patient had tried 6 lines of chemotherapy, yet he died within 8 months of his colon cancer diagnosis.

Further research is warranted to characterize macroscopic patterns, prognostication, and treatment plans for patients with SRCC of the colon with multiple sites of metastasis, such as the stomach and small intestine.

## DISCLOSURES

Author contributions: CK Tom: conception and design, acquisition, analysis, interpretation, drafting the work, final approval of the version to be published, agreement to be accountable for all aspects of the work. N. Placone: conception and design, analysis, interpretation, reviewing the work critically, final approval of the version to be published, agreement to be accountable for all aspects of the work. E. Yung: acquisition, reviewing the work critically, final approval of the version to be published, agreement to be accountable for all aspects of the work. A. Shaker: conception and design, reviewing the work critically, final approval of the version to be published, agreement to be accountable for all aspects of the work and is the article guarantor.

Financial disclosure: None to report.

Previous presentation: This case report has been accepted to the ACG 2023 Annual Scientific Meeting for poster presentation on October 2023; Vancouver, Canada.

Informed consent was obtained from the patient’s next of kin for this case report.
